# Outcomes of Cerebral Venous Thrombosis in Patients With Myeloproliferative Neoplasms from a U.S. Nationwide Hospitalization Study

**DOI:** 10.1002/jha2.70188

**Published:** 2025-12-26

**Authors:** Nikhil Vojjala, Raj Shah, Viktoriya Gibatova, Lakshmi Kattamuri, Shubham Agrawal, Geetha Krishnamoorthy, Rushad Patell

**Affiliations:** ^1^ Department of Internal Medicine Trinity Health Oakland Hospital Pontiac Michigan USA; ^2^ Wayne State University School of Medicine Detroit Michigan USA; ^3^ Department of Internal Medicine University of Kansas Wichita Kansas USA; ^4^ Ross University School of Medicine Bridgetown Barbados; ^5^ Department of Internal Medicine Texas Tech University Health Sciences Center El Paso El Paso Texas USA; ^6^ Department of Hematology Oncology University of Minnesota Minneapolis Minnesota USA; ^7^ Division of Hematology and Hematologic Malignancies Beth Israel Deaconess Medical Center Boston Massachusetts USA; ^8^ Harvard Medical School Boston Massachusetts USA

1

Dear Editor;

Cerebral venous thrombosis (CVT) is relatively rare, accounting for approximately 0.5%–3% of all strokes, but it can be associated with severe intracranial complications [1]. Approximately 10%–20% of survivors experience long‐term functional disability, often due to persistent neurological deficits [2]. Myeloproliferative neoplasms (MPNs), including polycythemia vera (PV), essential thrombocythemia (ET), and primary myelofibrosis (PMF), are characterized by a prothrombotic state driven by clonal myeloid cell proliferation and underlying driver mutations. Patients with MPN are at substantially increased risk for arterial and venous thrombosis, including thrombotic events in atypical locations such as splanchnic vein thrombosis or CVT [3]. An underlying MPN has been reported in up to 4%–5% of patients with CVT [4]. Moreover, patients with MPN who develop CVT may be linked with higher rates of recurrent thrombosis compared to other venous thrombosis despite anticoagulation, suggesting even higher prothrombotic risk [5]. We aimed to leverage a national database to investigate the impact of MPN on in‐hospitalization outcomes in patients with CVT.

We conducted a retrospective cohort study using the Nationwide Inpatient Sample (NIS) Database from January 1, 2018 to November 30, 2021. The NIS is an administrative database developed by the Agency for Healthcare Research and Quality for the Healthcare Cost and Utilization Project. This large, publicly available database contains de‐identified patients and hospital‐level information on approximately 35 million hospital discharges [6]. The study protocol was approved by the Institutional Review Board at Trinity Health Oakland Hospital/Wayne State University School of Medicine.

All adult patients (≥ 18 years) with primary discharge diagnosis of (I10‐DX1) CVT were included in the study. CVT admissions were identified using the International Classification of Diseases, 10th Revision, Clinical Modification (ICD‐10‐CM) I676, I636, and G08 [7]. Subsequently, MPNs were identified as a secondary diagnosis (I10‐DX2) for ET, PV, and PMF. Medical comorbidities, including hypertension, Type 2 diabetes mellitus, dyslipidemia, obesity, coronary artery disease, atrial fibrillation, congestive heart failure, chronic liver disease, chronic kidney disease, primary thrombophilia, antiphospholipid antibody syndrome, and transient ischemic attack, were identified as additional secondary diagnoses (I10‐DX2) using coding algorithms (Table S1). In addition, several patient‐level (age, sex, race, primary payer [Medicare, Medicaid, private insurance, and uninsured]) and hospital‐level (bed size [small, medium, and large], teaching status, location [rural or urban], and geographic region [Northeast, Midwest, West, or South]) variables were also extracted.

The primary outcome was in‐hospital mortality. Key secondary outcomes included concomitant non‐CVT venous thromboembolic events (deep venous thrombosis [DVT], pulmonary embolism [PE]). Other outcomes were health care utilization metrics such as length of stay (LOS), total hospitalization charge, and intensive care unit (ICU) utilization. ICU admissions were derived from procedural ICD‐10‐CM codes for either one of: (1) continuous invasive mechanical ventilation during hospitalization, (2) cardiopulmonary resuscitation > 24 h from patient's death, or (3) central venous catheterization [8].

Analysis was performed using Stata 90 software version 18.0 (Stata Corporation, College Station, TX, USA). Variables in the study were divided into continuous and categorical variables. Univariate analyses for between‐group comparisons for categorical variables used the Rao–Scott chi‐square test. Adjusted Wald's test was used for comparing the continuous variables, like LOS and total hospitalization charges. Subsequently, multivariate logistic regression for categorical variables with a binary outcome and linear regression for continuous variables were carried out to derive predictors of mortality. We conducted a secondary analysis with propensity score matching (PSM) to confirm the results obtained by multivariate analysis on an unmatched sample. A 1:1 nearest neighbor PSM without replacement algorithm was used. All the variables included in the unmatched logistic regression were applied to the matched sample, applying a caliper width of 0.2. Compared mortality rates between the groups by a multivariable regression model adjusted for demographics, hospital characteristics, and comorbidities. Further, we generated 224 matched hospitalizations (112 in each group) and compared mortality rates between the groups, including a multivariable regression model adjusted for demographics, hospital characteristics, and comorbidities.

Of 36,639 hospitalizations with CVT between 2018 and 2021, an MPN diagnosis was found in 575 (1.5%) patients. Among patients with MPN, ET was most prevalent (71%, *n* = 410), followed by PV (22.6%, *n* = 120) and PMF (4.3%, *n* = 25). The mean age (± SD) for patients without MPN was 51 (± 0.23) years, compared to 50 (± 1.72) years for the group with MPN. Overall, 56% (*n* = 20,484) were female, and the majority (89%, *n* = 32,689) were admitted to urban teaching hospitals, with most admissions occurring in facilities with large bed sizes (68%, *n* = 24,934). Across the entire cohort of CVT hospitalizations, the most prevalent comorbidities were hypertension (36%, *n* = 13,230), dyslipidemia (23%, *n* = 8275), and Type 2 diabetes mellitus (17%, *n* = 6245) (Table 1).

**TABLE 1:** Demographics, in‐hospital mortality, and hospital utilization metrics of cerebral venous thrombosis hospitalizations stratified by the presence of myeloproliferative neoplasms.

Baseline characteristics	Overall CVT (*n* = 36,639)	CVT without MPN (*n* = 36,064)	CVT with MPN (*n* = 575)	*p* value
Age, mean (± SD)	51.14 (± 0.22)	51.16 (± 0.23)	49.92 (± 1.72)	0.471
Sex				
Male	16,155 (44%)	15,835 (44%)	320 (56%)	0.012
Female	20,484 (56%)	20,229 (56%)	255 (44%)	
Race				
White	64%	64%	65%	0.461
African American	16%	16%	20%	
Hispanic	12%	12%	9%	
Other	7%	7%	6%	
Comorbidities				
Hypertension	13,230 (36%)	12,985 (36%)	245 (43%)	0.134
Smoking, active	10,240 (28%)	10,055 (28%)	185 (32%)	0.301
Dyslipidemia	8275 (23%)	8165 (23%)	110 (19%)	0.362
Obesity	6135 (17%)	6040 (17%)	95(17%)	0.254
Type 2 DM	6245 (17%)	6170 (17%)	75 (13%)	0.127
Atrial fibrillation	2595 (7%)	2575 (7%)	20 (3%)	0.193
CAD	2745 (7%)	2720 (8%)	25 (4%)	0.148
CHF	2120 (6%)	2105 (6%)	15 (3%)	0.941
Charlson Comorbidity Index				
0	21%	21%	17%	0.543
1	30%	30%	28%	
2	14%	14%	27&	
> 3	34%	34%	37%	
Thrombophilia				
Primary thrombophilia^a^	3187 (8.7%)	3135 (8.6%)	52 (9.0%)	0.422
APS syndrome	420 (1%)	415 (1%)	—	0.779
Primary expected payer				0.601
Medicare	11,340 (31%)	11,175 (31%)	165 (29%)	
Medicaid	7230 (20%)	7085 (20%)	145 (25%)	
Private insurance	14,525 (40%)	14,320 (40%)	205 (36%)	
Self‐pay (uninsured)	2075 (6%)	2045 (6%)	30 (5%)	
Others	1410 (3%)	1380 (3%)	30 (5%)	
Location				0.282
Northeast	7035 (19%)	6935 (19%) 7450 (21%) 13,155 (36%) 8524 (24%)	100 (17%)	
Midwest	7600 (21%)	6935 (19%) 7450 (21%) 13,155 (36%) 8524 (24%)	150 (26%)	
South	13,325 (36%)	6935 (19%) 7450 (21%) 13,155 (36%) 8524 (24%)	170 (30%)	
West	8679 (24%)	6935 (19%) 7450 (21%) 13,155 (36%) 8524 (24%)	155 (27%)	
Median house income				0.391
1st quartile	9305 (26%)	9170 (26%)	135 (24%)	
2nd quartile	9095 (25%)	8985 (25%)	110 (20%)	
3rd quartile	9415 (26%)	9240 (26%)	175 (31%)	
4th quartile	8160 (23%)	8020 (23%)	140 (25%)	
In‐hospitalization mortality	2165 (6%)	2130 (6%)	35 (6%)	0.934
Concomitant VTE				
Pulmonary embolism	1175 (3%)	1150 (3%)	25 (4%)	0.482
DVT	1500 (4%)	1465 (4%)	35 (6%)	0.271
ICU admission rates^b^	7805 (21.3%)	7665 (21.2%)	140 (24.3%)	0.421
Mean LOS	9.19 days	9.11 days	11.52 days	0.061
Mean total hospital charges	$149,784.7	$144,979.7	$163,044.8	0.364
Discharge disposition				0.672
Home	55%	55%	55%	
Short‐term hospital	5%	5%	7%	
Skilled nursing unit	24%	24%	26%	
Home health	15%	15%	12%	

*Note*: Univariate *p* value is calculated between MPN versus non‐MPN groups.

Abbreviations: APS, antiphospholipid antibody syndrome; CAD, coronary artery disease; CCI, Charleston Comorbidity Index; CHF, congestive heart failure; CI, confidence interval; CVT, cerebral venous thrombosis; DVT, deep venous thrombosis; ICU, intensive care unit; LOS, length of stay; MPN, myeloproliferative neoplasms; Type 2 DM, Type 2 diabetes mellitus; VTE, venous thromboembolism.

^a^ Primary thrombophilia disorders included in the analysis were prothrombin gene mutation, antithrombin 3 deficiency, protein C deficiency, protein S deficiency, activated protein C resistance, and any other primary thrombophilia not specified or classified.

^b^ Either mechanical ventilation, central line placement, or cardiopulmonary resuscitation that occurred more than 24 h before death, or a combination of them, are used as surrogates for identifying ICU admissions in the index study.

The overall in‐hospital mortality rate was 6% (*n* = 2165) and was consistent across both groups, with 35 deaths (6%) recorded among patients with MPN and 2130 deaths (6%) among those without (Table 1). In the multivariate logistic regression analysis, an MPN diagnosis was not associated with any difference in in‐hospital mortality (OR: 1.12, 95% CI: 0.50–2.49, *p* = 0.769) (Table 2). Comorbid conditions such as congestive heart failure (CHF) demonstrated substantially increased odds of mortality (OR: 2.10, 95% CI: 1.41–3.11, *p* < 0.001). Patients with concomitant non‐CVT VTE events like DVT (OR: 1.61, 95% CI: 1.06–2.47, *p* = 0.026) and PE (OR: 1.76, 95% CI: 1.10–2.82, *p* = 0.017) were associated with increased mortality.

**TABLE 2:** Multivariate logistic regression analysis for factors associated with in‐hospital mortality in the overall cohort (*n* = 36639).

Patient features	Odds ratio	95% CI	*p *value
MPN	1.12	(0.50, 2.49)	0.761
Age^a^	1.01	(1.01, 1.02)	0.001
Female	0.69	(0.56, 0.85)	0.001
Income in quartiles			
2nd quartile	0.77	(0.59, 1.01)	0.063
3rd quartile	0.56	(0.42, 0.74)	0.001
4th quartile	0.57	(0.42, 0.78)	0.001
Hospital bed size^c^			
Medium	1.03	(0.67, 1.58)	0.878
Large	1.52	(1.05, 2.20)	0.025
Loc/hosp teaching^d^			
Urban/nonteaching	1.94	(0.73, 5.14)	0.179
Urban teaching	2.43	(0.98, 5.98)	0.053
Region			
Midwest	0.93	(0.68, 1.26)	0.650
South	0.96	(0.72, 1.28)	0.795
West	0.97	(0.71, 1.32)	0.863
Comorbidities			
HTN	0.98	(0.77, 1.24)	0.882
Type 2 DM	1.04	(0.79, 1.37)	0.758
Dyslipidemia	0.62	(0.47, 0.82)	0.001
Atrial fibrillation	1.32	(0.91, 1.93)	0.136
CAD	0.83	(0.56, 1.22)	0.362
CHF	2.10	(1.41, 3.11)	0.001
CKD	1.12	(0.58, 2.18)	0.717
Nephrotic syndrome	1.92	(0.27, 13.5)	0.511
Obesity	0.76	(0.55, 1.04)	0.092
Smoking	0.46	(0.35, 0.60)	0.000
APD	0.63	(0.19, 2.13)	0.468
TIA	0.48	(0.75, 3.11)	0.446
Thrombophilia	0.64	(0.41, 1.04)	0.052
Elixhauser Index^b^	1.29	(1.21, 1.37)	0.001
Concomitant DVT	1.62	(1.06, 2.47)	0.026
Concomitant PE	1.76	(1.10, 2.82)	0.017

Abbreviations: APS, antiphospholipid antibody syndrome; CAD, coronary artery disease; CCI, Charlson Comorbidity Index; CHF, congestive heart failure; CI, confidence interval; CVT, cerebral venous thrombosis; DVT, deep venous thrombosis; LOS, length of stay; MPN, myeloproliferative neoplasms; Type 2 DM, Type 2 diabetes mellitus; VTE, venous thromboembolism.

^a^ For age, we used a cut‐off of 49 years, which is the mean age of the MPN with the CVT group.

^b^ For the Elixhauser Index, a cutoff of 15 is used.

^c^ The small hospital bed size is the comparator used for determining bed size. The bed size cut‐off varies according to the type of hospital setting (rural vs. urban, non‐teaching vs. urban teaching setting). For a rural setting, small bed size is 1–49 beds, medium bed size is 50–99 beds, and large bed size is more than 100 beds. For urban, non‐teaching settings, small bed size is 1–99 beds, medium bed size is 100–199 beds, and large bed size is more than 200 beds. For urban teaching hospitals, small bed size is 1–249 beds, medium bed size is 250–499 beds, and large bed size is more than 500 beds.

^d^ Among regions, the Northeast is used as the comparator for the rest of the areas.

A total of 224 hospitalizations (112 in each group) were included in the PSM analysis to derive predictors of mortality. After PSM, the mean bias decreased from 10.8% in the unmatched sample to 5.7% in the matched sample, representing a 46.9% reduction (Figure S1). There was no significant difference in mortality in MPN versus non‐MPN patients in both univariable (6.2% vs. 3.5%, *p* = 0.35) and multivariable regression models (OR 1.80, 95% CI: 0.51–6.33) (Table S2).

The mean LOS was numerically higher in the MPN group compared to those without MPN (11.52 days vs. 9.11 days [*p* = 0.06]). The mean hospitalization charges were also numerically higher among the MPN group, amounting to $163,044.8 compared to $144,979.7 for patients without MPN (*p *= 0.301). Overall, 21.3% (*n *= 7805) had ICU admission during index hospitalization, and rates were not statistically significant between the two groups (*p *= 0.423). Of note, 4% (*n* = 1500) and 3% (*n* = 1175) had concomitant DVT and PE, respectively, and the proportion of these events was not significantly different between the groups (*p* = 0.278).

We report an overall prevalence rate of 1.5% for MPN among CVT hospitalizations, in line with other large international CVT cohorts like the International Study on Cerebral Vein and Dural Sinus Thrombosis (ISCVT) (2.9%) and French Prospective Cohort of Cerebral Venous Thrombosis (FPCCVT) (1.7%) [9]. The in‐hospital mortality rate among patients with CVT of 6% was concordant with prior reported mortality rates in the real world, ranging from 4% to 10% [4, 5]. The mortality was not impacted by underlying MPN diagnosis; like a subgroup analysis of the FPCCVT cohort that found there was no significant difference in the mortality observed in the JAK2V617F‐positive cohort versus those without JAK2 mutation (*n* = 216, including nine patients with JAK2 mutation) [10].

Although prior studies have identified prognostic variables in patients with CVT, there have been limited systematic assessments of the impact of an underlying MPN diagnosis in patients with CVT, despite the well‐recognized association of MPN with CVT [4]. The key predictors of in‐hospital mortality identified in our study include comorbidities such as heart failure, concomitant VTE during the index hospitalization, and a higher Elixhauser Comorbidity Index. Underlying MPN was not associated with increased mortality or ICU use in hospitalized adults with CVT. Additionally, in our study, the higher LOS and total hospitalization charges for patients with MPN would be due to higher acuity, more hemorrhagic complications, and subsequent intensive medical interventions, which would need to be explored in prospective multicenter studies.

The strengths of this study are the large sample size of atypical site thrombosis in patients with MPN. Second, population‐level assessment allows generalizability of findings. However, although the NIS is designed to generate a representative dataset, identification of cohorts relies on billing codes and thus could lead to biases from misclassification. To overcome this, we used previously validated algorithms, when available, to classify the cohorts. In addition, the dataset did not allow us to capture certain potentially influential variables, such as hemorrhagic complications, treatment patterns, and the functional status of the patients. We were also unable to assess the impact of the type of driver mutation and or allele frequency, as these data are not available, although they can be important prognostic predictors in patients with MPN. Similarly, we were also not able to determine the MPN‐directed treatment protocols used and their bearing on the outcomes of the hospitalizations.

The relatively small number of MPN‐associated CVT hospitalizations (*n* = 575) and mortality events (*n* = 35), particularly for PMF (*n *= 25), contributed to wide confidence intervals and point to limited statistical power. Thus, the non‐significant mortality difference between the groups in the PSM analysis should be interpreted as statistically inconclusive given the small, matched sample size (*n* = 224) and moderate bias reduction (46.9%) following PSM. Moreover, due to limitations inherent to the dataset, the lack of information about anticoagulation regimens, duration of therapy, or MPN‐directed treatments such as cytoreductive agents may explain the discrepancy in the rates of DVT/PE in our study compared to previous studies. These shortcomings, including granularity regarding driver mutation status, hemorrhagic complications, and long‐term outcomes, can be addressed in future large‐scale prospective studies.

This population‐level retrospective analysis of hospitalizations for CVT provides valuable insights. Although an MPN diagnosis did not impact short‐term mortality, further investigation to better understand the long‐term implications of this condition in the context of CVT is warranted. In addition, this study highlights the need to refine risk stratification models and develop targeted interventions aimed at improving outcomes in this high‐risk patient population.

## Funding

The authors have nothing to report.

## Conflicts of Interest

Rushad Patell reports consultancy from Merck Research, outside of current work. The other authors declare no conflicts of interest.

## Supporting information




**Supporting Table 1**: Billing codes ICD 10 codes of all the primary diagnoses and secondary diagnoses (comorbid conditions) used in the study.
**Supplementary Table 2**: Part A: Propensity Score Matching with Standardized Mean Differences of the bias before and after matching.
**Supplementary Figure 1**: Bland‐Altman plot for bias estimation in unmatched and matched cohorts

## Data Availability

Data is available publicly with the Health Care Cost and Utilization Project (HCUP) central distributor.
