# Supplementary material for: Outcomes of Cerebral Venous Thrombosis in Patients With Myeloproliferative Neoplasms from a U.S. Nationwide Hospitalization Study
Source: EJHaem. 2025 Dec 26;7(1):e70188. doi: 10.1002/jha2.70188 (PMC12742990; doi:10.1002/jha2.70188)
Supplement: Supplementary file 1 — Supporting Table 1: Billing codes ICD 10 codes of all the primary diagnoses and secondary diagnoses (comorbid conditions) used in the study. Supplementary Table 2: Part A: Propensity Score Matching with Standardized Mean Differences of the bias before and after matching. Supplementary Figure 1: Bland‐Altman plot for bias estimation in unmatched and matched cohorts [file JHA2-7-e70188-s001.docx]

**Supplementary Table 1**

**Billing codes ICD 10 codes of all the primary diagnoses and secondary diagnoses (comorbid conditions) used in the study:**

| **Diagnosis** | **ICD 10 codes** |
| --- | --- |
| Cerebral Venous Sinus Thrombosis | I676, I636, and G08. |
| Myeloproliferative Neoplasm | D473, D45 and D7581 |
| Type 2 Diabetes mellitus | E10, E11 |
| Hypertension | I10 |
| Dyslipidemia | E785 |
| Obesity | E66 |
| Coronary artery disease | I25 |
| Congestive heart failure | I50 |
| Atrial fibrillation | I48 |
| Chronic kidney disease | N189 |
| Nephrotic syndrome | N04 |
| Budd Chiari syndrome | I820 |
| Transient ischemic attack | G459 |
| Antiphospholipid antibody syndrome | D6861 |
| Primary Thrombophilia | D68.5 (D68.51: Activated Protein C resistance, D68.52: Prothrombin Gene Mutation, D68.59: Other primary thrombophilia which includes Antithrombin 3 deficiency, Hypercoagulable state NOS, Primary Hypercoagulable state Not specific or classified, Primary Thrombophilia NEC, Protein C deficiency, Protein S deficiency, Thrombophilia NOS) |
| Deep venous thrombosis | I82629, I82419, I82623, I824Z9, I82623, I82621, I824Y9, I82621, I8240, I82622, I82623, I8290, I82629, I82402, I82403, I82421, I82413, I82411, I824Y2, I82623, I82433, I82493, I82491, I82433, I82493, I2491, I82443, I82412, I82441, I82A12, I82621, I82622, I82449, I82A19, I824Y3, I82629, I82429, I82A11, I824Y1, I82422, I82409, I82401, I824Z2, I82423, I82442, I82431, I824Z3, I82492, I824Z1 |
| Pulmonary embolism | I2609, I2699, I2601, I2692, I2690, I2602 |
| Smoking, active | F17200, F17201, F17203, F17208, F17200, F17209, F17210, F17211, F17213, F17218, F17219, F17220, F17221, F17223, F17228, F17229, F17290, F17291, F17293, F17298, F17299 |

**Supplementary Table 2**

**Part A: Propensity Score Matching with Standardized Mean Differences of the bias before and after matching**

| **Variable** | **Unmatched** | **CVT with MPN** | **CVT without MPN** | **%Bias** | **%Bias reduction** | **P-value** |
| --- | --- | --- | --- | --- | --- | --- |
|  | **Matched** |  |  |  |  |  |
| **Age** | U | 49.28 | 51.05 | -9.5 | 48.9 | 0.32 |
|  | M | 49.28 | 48.38 | 4.9 |  | 0.71 |
| **Female Gender** | U | 45.5% | 56.3% | -21.8 | 83.5 | 0.02 |
|  | M | 45.5% | 47.3% | -3.6 |  | 0.79 |
| **HTN** | U | 43.7% | 36.0% | 15.8 | 53.9 | 0.09 |
|  | M | 43.7% | 40.1% | 7.3 |  | 0.59 |
| **Type 2 DM** | U | 13.3% | 17.0% | -10.3 | 100.0 | 0.30 |
|  | M | 13.3% | 13.3% | 0.0 |  | 1.00 |
| **Dyslipidemia** | U | 19.6% | 22.6% | -7.2 | -111.2 | 0.45 |
|  | M | 19.6% | 13.3% | 15.3 |  | 0.21 |
| **Atrial fibrillation** | U | 35.7% | 7.1% | -15.9 | 25.2 | 0.14 |
|  | M | 35.7% | 0.8% | 11.9 |  | 0.17 |
| **CAD** | U | 4.4% | 7.5% | -12.9 | 12.7 | 0.22 |
|  | M | 4.4% | 7.1% | -11.3 |  | 0.39 |
| **CHF** | U | 2.6% | 5.8% | -15.6 | 100.0 | 0.15 |
|  | M | 2.6% | 2.6% | 0.0 |  | 1.00 |
| **CKD** | U | 0.8% | 1.7% | -7.5 | 100.0 | 0.49 |
|  | M | 0.8% | 0.8% | 0.0 |  | 1.00 |
| **Obesity** | U | 16.0% | 16.9% | -2.3 | -112.3 | 0.81 |
|  | M | 16.0% | 17.8% | -4.8 |  | 0.72 |
| **Smoking** | U | 33.0% | 27.9% | 11.0 | 64.7 | 0.23 |
|  | M | 33.0% | 31.2% | 3.9 |  | 0.77 |
| **Thrombophilia** | U | 9.8% | 8.6% | 3.9 | -56.9 | 0.67 |
|  | M | 9.8% | 8.0% | 6.1 |  | 0.64 |
| **CCI** | U | 1.7 | 1.6 | 12.9 | 15.9 | 0.17 |
|  | M | 1.7 | 1.8 | -10.9 |  | 0.41 |

*CVT: Cerebral Venous Thrombosis, CI: Confidence Interval, CCI: Charleston Comorbidity index, CHF: Congestive heart failure, CAD: Coronary Artery Disease, DVT: Deep Venous Thrombosis, LOS: Length of Stay, MPN: Myeloproliferative Neoplasms, Type 2 DM: Type 2 Diabetes Mellitus. Mean bias before matching is 10.8%, and after matching, it is 5.7%, indicating a 51% overall reduction of mean bias. Rubin’s B statistic dropped from 47.6 to 35.5.*

**Part B: Mortality rates on a propensity matched sample (n=224, 112 in each category)**

|  | **CVT with MPN** | **CVT without MPN** | **P-value** |
| --- | --- | --- | --- |
| Mortality rates | 6.2% | 3.5% | 0.35 |

**Part C: Multivariable Logistic Regression Analysis for Predictors of Mortality in propensity-matched cohort (n=224)**

| **Patient Features** | **Odds ratio** | **(95% CI)** | **P-value** |
| --- | --- | --- | --- |
| **MPN** | 1.8 | (0.50,6.33) | 0.360 |
| **Age** | 0.99 | (0.98, 1.00) | 0.190 |
| **Sex (Female: Male)** | 0.63 | (0.43,0.93) | **0.018** |
| **Income in Quartiles** | 1.13 | (0.95,1.35) | 0.153 |
| **Hospital Bed size** | 1.09 | (0.81,1.46) | 0.574 |
| **Loc/Hosp teaching** | 0.77 | (0.50,1.19) | 0.235 |
| **Region** | 0.99 | (0.83,1.18) | 0.910 |
| **Comorbidities**  HTN  Type 2 DM  Dyslipidemia  Atrial fibrillation  CAD  CHF  CKD  Obesity  Smoking  APD  Thrombophilia  CCI | 1.52  0.68  0.89  0.53  0.62  0.65  0.57  1.01  1.21  0.69  1.09  1.24 | (1.00,2.31)  (0.38,1.24)  (0.53,1.50)  (0.19,1.50)  (0.24,1.60)  (0.19,2.20)  (0.08,4.22)  (0.60,1.71)  (0.80,1.81)  (0.09,5.05)  (0.58,2.06)  (1.04,1.49) | **0.053**  0.210  0.672  0.234  0.322  0.491  0.583  0.964  0.369  0.714  0.793  **0.018** |
| **Concomitant VTE**  DVT  PE | 1.50  1.21 | (0.68,3.33)  (0.48,3.07) | 0.319  0.689 |

*APS: Antiphospholipid antibody syndrome, CVT: Cerebral Venous Thrombosis, CI: Confidence Interval, CCI: Charleston Comorbidity index, CHF: Congestive heart failure, CAD: Coronary artery disease, DVT: Deep Venous Thrombosis, LOS: Length of Stay, MPN: Myeloproliferative Neoplasms, Type 2 DM: Type 2 Diabetes Mellitus, VTE: Venous Thromboembolism. For continuous variables like age: we used the mean age of the MPN group, i.e., 49 years, as a cut-off and represented it as binary. Income Quartile 1 is compared to others. Among regions, the northeast is compared to other regions. Hospital bed size: Small size is used as a comparator.*

**Supplementary Figure 1: Bland-Altman plot for bias estimation in unmatched and matched cohorts**

**
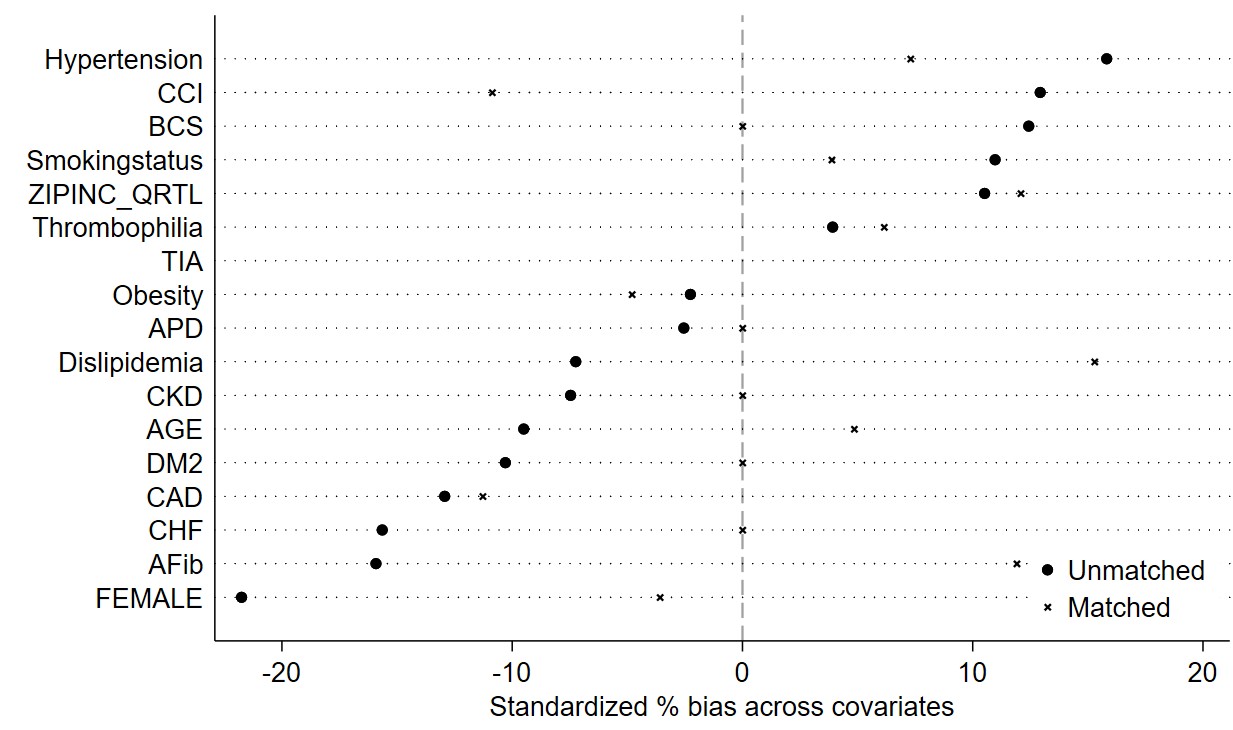
**

*APS: Antiphospholipid antibody syndrome, CVT: Cerebral Venous Thrombosis, A.fib: Atrial Fibrillation, CI: Confidence Interval, CCI: Charleston Comorbidity index, CHF: Congestive heart failure, CAD: Coronary artery disease, DVT: Deep Venous Thrombosis, LOS: Length of Stay, MPN: Myeloproliferative Neoplasms, Type 2 DM: Type 2 Diabetes Mellitus, VTE: Venous Thromboembolism.*
